# Systematic review on human resources for health interventions to improve maternal health outcomes: evidence from low- and middle-income countries

**DOI:** 10.1186/s12960-016-0106-y

**Published:** 2016-03-12

**Authors:** Zohra S. Lassi, Nabiha B. Musavi, Blerta Maliqi, Nadia Mansoor, Andres de Francisco, Kadidiatou Toure, Zulfiqar A. Bhutta

**Affiliations:** Division of Women and Child Health, Aga Khan University, Karachi, Pakistan; Robinson Research Institute, The University of Adelaide, Adelaide, South Australia Australia; Department of Maternal Newborn Child and Adolescent Health, World Health Organization, Geneva, Switzerland; Partnership for Maternal Newborn & Child Health, Geneva, Switzerland; Center of Excellence in Women and Child Health, Aga Khan University, Karachi, Pakistan; Centre for Global Child Health, The Hospital for Sick children, Toronto, Canada

**Keywords:** Human resources for health, Maternal mortality, Skilled birth attendant, Low- and middle-income countries

## Abstract

There is a broad consensus and evidence that shows qualified, accessible, and responsive human resources for health (HRH) can make a major impact on the health of the populations. At the same time, there is widespread recognition that HRH crises particularly in low- and middle-income countries (LMICs) impede the achievement of better health outcomes/targets. In order to achieve the Sustainable Development Goals (SDGs), equitable access to a skilled and motivated health worker within a performing health system is need to be ensured. This review contributes to the vast pool of literature towards the assessment of HRH for maternal health and is focused on interventions delivered by skilled birth attendants (SBAs). Studies were included if (a) any HRH interventions in management system, policy, finance, education, partnership, and leadership were implemented; (b) these were related to SBA; (c) reported outcomes related to maternal health; (d) the studies were conducted in LMICs; and (e) studies were in English. Studies were excluded if traditional birth attendants and/or community health workers were trained. The review identified 25 studies which revealed reasons for poor maternal health outcomes in LMICs despite the efforts and policies implemented throughout these years. This review suggested an urgent and immediate need for formative evidence-based research on effective HRH interventions for improved maternal health outcomes. Other initiatives such as education and empowerment of women, alleviating poverty, establishing gender equality, and provision of infrastructure, equipment, drugs, and supplies are all integral components that are required to achieve SDGs by reducing maternal mortality and improving maternal health.

## Background

Maternal health is one of the main global health challenges in which least progress was witnessed in the year 2015 [1]. Hence, greater progress is required to meet the newly developed Sustainable Development Goals (SDGs) by ensuring equitable access to a skilled and motivated health worker within a performing health system. Less than 1% of the annual maternal deaths occur in the developed world, while a large proportion of these occur in low- and middle-income countries (LMICs). Further, for every woman dying, at least 30 others suffer complications which often end up being long-term and devastating which includes infertility and damage to the reproductive organs. It is widely agreed that there is not a single straightforward intervention, which can bring significant decrease in maternal mortality, but it can only be addressed by strengthening and providing an efficient health system with the provision of trained health workers being its key component [2–4]. The shortage of health workers was the most significant constraint noticed for not attaining the three health-related Millennium Development Goal (MDG) targets by many countries [3, 5–8].

Unfortunately, the workforce is distributed unevenly [9, 10]. Asia, a continent with half of the world’s population, has access to only 30% of the world’s health professionals [11]. Africa, with the highest burden of disease, has access to only 1% of the world’s health professionals [11]. Whereas America which has 10% of the global burden of disease has approximately 40% of the world’s health professionals [11]. The scenario within each country also shows asymmetry in the distribution of health professionals with low number of professionals in the rural compared to urban areas [12, 13]. Apart from mal-distribution, many countries face difficulties in recruiting and retaining health professionals. Insufficient number of medical schools, low salaries of the existing health workforce, poor working conditions, lack of supervision, low morale and motivation, and lack of infrastructure are few prominent causes for the loss of health professionals, where they immigrate to wealthier countries [14–18].

The shortage of emergency obstetric care (EmOC) and surgical services in LMICs over the last decade has attracted substantial attention [19–22]. In response, governments, health organizations, and communities have taken actions to address human resources for health (HRH) needs. There is a substantial evidence that highlights initiatives and innovative actions which have increased efficiency in utilizing existing human resources, including team approaches for delivery of intervention, multi-tasking, task shifting and sharing, and increased involvement by the communities [23]. However, most of them are implemented on a small scale or at limited capacity. In this context, it is important to realize that there is immense need for better planning, distribution, and management of limited human resources to address SDGs and strong need for formative evidence learned through lessons towards achieving this goal. This review has focused on the impact of HRH interventions for maternal health delivered by skilled birth attendants (SBAs) [24, 25] (Table 1). We have also derived lessons, identified research gaps, and formulated recommendations based on the studies from LMICs.

**Table 1:** Definition of skilled birth attendant and skilled birth attendance

Skilled birth attendant	Skilled birth attendance
A joint WHO/ICM/FIGO statement, endorsed by UNFPA and the World Bank defines a skilled attendant as “an accredited health professional, such as a midwife, doctor or nurse, who has been educated and trained to proficiency in the skills needed to manage normal (uncomplicated) pregnancies, childbirth and the immediate postnatal period, and in the identification, management and referral of complications in women and newborns” [79].	Skilled attendance is the process by which a pregnant woman and her infant are provided with adequate care during pregnancy, labor, birth, and the postpartum and immediate newborn periods, whether the place of delivery is the home, health center, or hospital. In order for this process to take place, the attendant must have the necessary skills and must be supported by an enabling environment at various levels of the health system, including a supportive policy and regulatory framework; adequate supplies, equipment, and infrastructure; and an efficient and effective system of communication and referral/transport [80, 81].

Source: DFID 2005 [82]

## Methods

The review derived evidence from randomized controlled trials (RCTs), quasi-RCTs, and prospective before/after and cohort studies on SBAs working at the national, provincial, district, and community levels (home, community or referral facility interventions). Studies were included if (a) they implemented any HRH interventions in management system, policy, finance, education, partnership, and leadership; (b) those were related to SBAs; (c) have reported outcomes related to maternal health such as changes in morbidity, mortality, coverage, or other interrelated outcomes; (d) were conducted in LMICs [26]; and (e) were written in English. Studies in which traditional birth attendants and/or community health workers were trained were excluded.

**Fig. 1:**
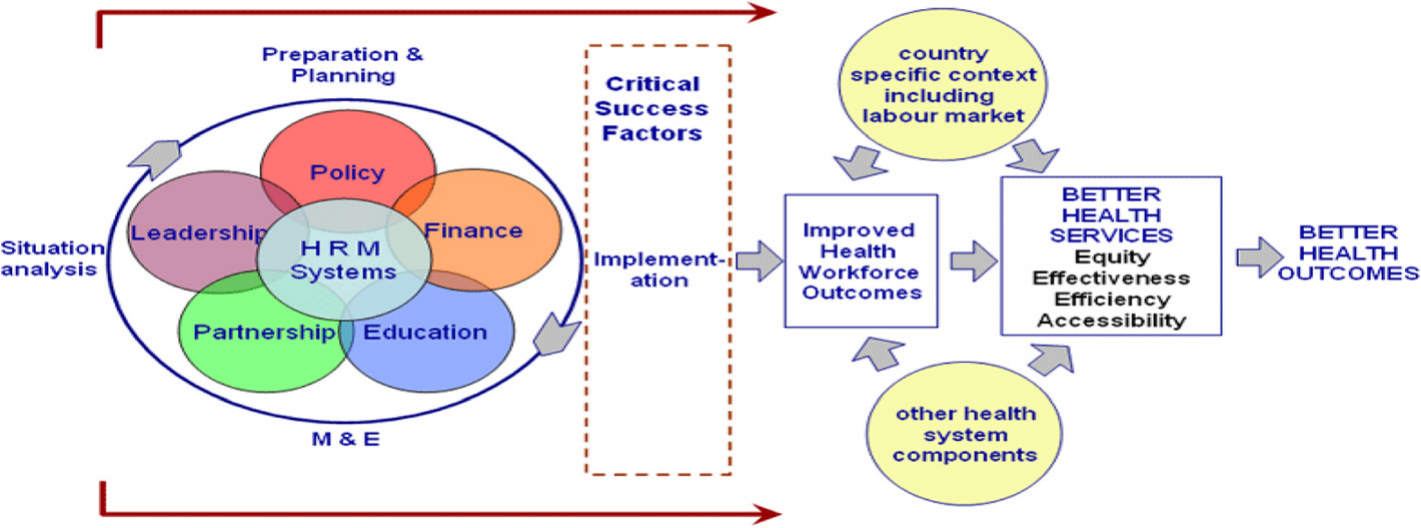
HRH action framework

The search strategy included PubMed articles published from January 2000 to December 2015. The reason to select this time period was to evaluate what progress has been made since the 2000 deadline of the Alma Ata health for all and what improvements followed on the agreement on MDGs. The HRH Global Resource Centre was also searched to access the available studies. Detailed examination of cross-references and bibliographies of identified studies was also performed to identify additional sources of information. The following search strategy was primarily used. [(“health worker*” OR “health care worker*” OR “health professional” OR “health personnel” OR doctor* OR nurse* OR physician* OR midwi* OR “nurse midwi*” OR “skilled birth attendant*”) AND (training OR education OR curriculum OR teaching OR learning OR “patient centered care” OR “patient focused care” OR “staff development” OR medicine OR “postgraduate training” OR “diploma training” OR recruitment OR attraction OR deployment OR employment OR personnel selection OR incentive OR reward OR “cash award”)]. The quality of RCTs and quasi-RCTs was assessed using the Cochrane methods [27]; however, the quality of prospective studies/pre-post trials was assessed using the criteria adopted from Loevinsohn (Table 2) [28]. To assess the different dimensions of the HR planning and management spectrum, this review used the HRH action framework (Fig. 1) as defined by WHO [29]. The framework has six action fields, each of which has several areas of intervention. For a better response to HRH crises, each of the six action fields needs to be addressed. These are described in Table 3.

**Table 2:** Quality assessment criteria for pre-post studies without control arm

Study features^a^	Assessment
1. Study based on explicit theory	Yes/no/unclear
2. Adequate description of how intervention strategy adapted to local conditions	Yes/no/unclear
3. Example given of materials or process	Yes/no/unclear
4. Adequate description of resources required to carry out interventions	Yes/no/unclear
5. Measure outcome before and after intervention	Yes/no/unclear
6. Measurement method same before and after	Yes/no/unclear
7. Period between education and outcome more than 1 year	Yes/no/unclear
8. Author claimed positive results for interventions	Yes/no/unclear
9. Paper included discussion of possible biases and caveats (or limitations)	Yes/no/unclear
10. Paper included *P* values or confidence interval	Yes/no/unclear
11. Analysis employed some form of modeling such as regression	Yes/no/unclear
12. Exposure to intervention monitored	Yes/no/unclear

^a^Adopted from Loevinsohn [28]

**Table 3:** HRH intervention assessed

1. Management systems *-Personnel systems*: workforce planning (including staffing norms), recruitment, hiring, and deployment *-Work environment and conditions*: employee relations, workplace safety, job satisfaction, and career development Work environment and conditions: employee relations, workplace safety, job satisfaction, and career development -HR information system integration of data sources to ensure timely availability of accurate data required for planning, training, appraising, and supporting the workforce -Performance management: performance appraisal, supervision, and productivity 2. Education *-Pre-service* education tied to health needs -*In-service* training (e.g., distance and blended, continuing education) - Capacity of training institutions -Training of community health workers and non-formal care providers. 3. Policy -Professional standards, licensing, and accreditation -Authorized scopes of practice for health cadres -Political, social, and financial decisions and choices that impact HRH -Employment law and rules for civil service and other employers	4. Leadership -Support HRH champions and advocates - Capacity for leadership and management at all levels -Capacity to lead multi-sector and sector-wide collaboration - Strengthening professional associations to provide leadership among their constituencies 5. Partnership -Mechanisms and processes for multi-stakeholder cooperation (interministerial committees, health worker advisory groups, observatories, donor coordination groups) - Public-private sector agreements -Community involvement in care, treatment, and governance of health services 6. Finance -Setting levels of salaries and allowances -Budgeting and projections for HRH intervention resource requirements including salaries, allowances, education, incentive packages, etc. -Increasing fiscal space and mobilizing financial resources (e.g., government, Global Fund, PEPFAR, donors) -Data on HRH expenditures (e.g., National Health Accounts)

Source: Capacity Project [29]

## Results

**Fig. 2:**
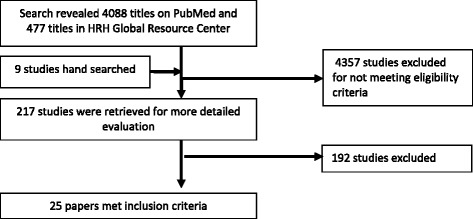
Conceptual framework of HRH interventions for improved maternal outcomes

The defined search strategy identified 4565 studies. Of these, 217 were retrieved for full-text review; however, only 25 papers passed the eligibility criteria for inclusion (Fig. 2). Studies were grouped and analyzed according to different dimensions and components of HRH interventions. However, we found studies related to education, policies, and those with multiple combined interventions.

### Education (training/task shifting)

We identified 15 low- to moderate-quality studies. Of these, four were RCTs [30–33], eight were prospective (before/after) [34–41], two were quasi-experimental [42, 43], and one was a prospective cohort [44]. Most of these were from African and South East Asian regions (Table 4).

**Table 4:** Studies related to HRH interventions: training and task shifting

Study, year and type	Type of training/intervention	Duration of training	Time between intervention and evaluation	Task performed by	Trained by	Area of intervention observed	Other interventions/tasks	Effects of training/results	Cost of training	Quality of study
Dusitsin [30] Thailand RCT	Tubal ligation in healthy women	–	–	Midlevel health workers	–	Operating theaters	–	No difference was found between the groups in postoperative complications (RR 2.43; 95% CI, 0.64–9.22).	–	Selection = UR
										Performance and detection = UR
										Attrition = UR
										Reporting = LR
Eren [31] Philippines and Turkey controlled trial	Intrauterine device insertion by auxiliary nurse midwives	–	–	Auxiliary nurse midwives	–	Teaching hospitals	–	No difference was seen in those who were referred to a specialist after insertion of an intrauterine device (RR 0.93; 95% CI, 0.45–1.90).		Selection = UR
										Performance and detection = UR
										Attrition = UR
										Reporting = LR
Warriner [32] South Africa and Viet Nam RCT	Manual vacuum aspiration performed by a midlevel health worker, with a follow-up 10 to 14 days later.	–	–	Midwives and doctor’s assistants	–	Primary care	–	Manual vacuum aspiration was significantly greater with auxiliary nurse midwives.		Selection = LR
										Performance = LR
										Detection = UR
										Attrition = LR
										Reporting = LR
Warriner [33] Nepal RCT	Administration of early medical abortion	–	–	Certified nurses and auxiliary nurses	–	Primary care	Midlevel health workers had full responsibility for the management of each case.	There was no significant difference in the likelihood of an incomplete abortion between groups of patients managed by auxiliary nurse midwives and those managed by doctors (RR: 0.93; 95% CI, 0.45–1.90). Nor was the likelihood of a complication during (RR: 3.07; 95% CI, 0.16–59.1)—or an adverse event after (RR: 1.36; 95% CI, 0.54–3.40)	-	Selection = LR
										Performance = LR
										Detection = UR
										Attrition = LR
										Reporting = LR
Mekbib [34] Ethiopia prospective (before/after)	This training focused on life-saving procedures in obstetric emergencies (C-sections, hysterectomies including management of incomplete abortion, post abortion scare, and ectopic pregnancy).	3 rounds of training were conducted. Each for 3 months period	Interventions began in 1999, and the results were analyzed in 2001.	GPs, midwives, and other service providers in EmOC	Department of obs/gyne and master trainers	Gandhi Memorial hospital in Addis Ababa and Ambo hospital	Management and coordination	The total number of deliveries at hospital increased by 39.7% from the baseline when compared with the year 2001. Instrumental deliveries increased from 6% in 1998 to 23% in 2001. The CFR for 1999 was 7.2% based on 18 deaths and for 2001 was 4.6% based on 20 deaths.	Almost $100 000 was used	1Y, 2Y, 3Y, 4Y, 5Y, 6Y, 7Y, 8Y, 9N, 10N, 11U, 12Y
							Equipment, supplies, and drugs			
							Record keeping			
							Blood supply			
Djan [35] Ghana prospective (before/after)	Midwife was trained in vacuum extraction, manual removal or retained placenta, and suturing of episiotomies and lacerations. -MOs were trained to manage obstetric emergencies.	2 weeks training	Intervention implemented 1993 and 1994 and evaluated in 1995	Midwives and medical officers		Koforidua, Ghana, and tertiary hospital KATH	OT, blood bank	The number of women with complications coming increased from 26 in 1993 to 73 in 1995, and the proportion of these who were referred for treatment dropped 42–14%. Surgical obstetric procedures performed increased from 23 to 90. Midwives performed 32% manual removal, 58% vacuum extractions, and 98% episiotomy repairs. No death occurred.	US$ 30 000 but mostly for equipment and supplies	1Y, 2Y, 3Y, 4Y, 5Y, 6N, 7Y, 8Y, 9N, 10N, 11Y, 12Y
							Maternity refurbished			
							Revolving drug fund. Running water supply			
							Improving access and reducing delay to care			
Ifenne [36] Nigeria prospective (before/after)	In-house training of midwives and residents in principles and practices of EmOC		Intervention started in 1993, and results were analyzed on 1994 and 1995.	Midwives and residents		Ahmadu Bello University Teaching Hospital	-OT restored -Maternity ward renovated -Improved access and -reduced delay to care -Blood bank and drug pack system	Admission to treatment interval was reduced from 3.7 h to 1.6 h. Proportion of women treated in less than 30 min increased from 39% to 87%. CFR fell from 14% to 11%. The annual number of women with complication declined from 326 to 65.	US$ 135 000	1Y, 2Y, 3Y, 4Y, 5Y, 6U, 7Y, 8Y, 9N, 10N, 11U, 12Y
Kruk [37] Mozambique prospective (before/after)	2-year classroom-based instruction and 1-year internship	2–3 years	Training began in 1983/1984 and was evaluated in 2007	Nurses and medical assistants	Surgeons in Mozambique	Provincial hospitals		In 2002, 47 specialists and 53 AMOs performed 5 264 and 6 914 major obstetric surgeries, respectively.	The 30-year cost for obstetric surgery was $38.9 for AMOs and $144.1 for physicians. After doubling the salaries of AMOs lead to major difference in cost	1Y, 2Y, 3Y, 4Y, 5Y, 6U, 7Y, 8Y, 9Y, 10N, 11Y, 12Y
GHWA [38] Bangladesh prospective (before/after)	Training of MOs in obstetrics and anesthesia, nurses in midwifery, and laboratory technicians in safe blood transfusion. In 2003, a new 17-week competency-based training program, along with 1-year training on obs and gyne was introduced for MOs and nurses.	Training of MOs for 1 year. Training of nurses for 4 months. Laboratory technicians for 2 weeks.	Baseline figures were taken in 1999, and then, interventions were implemented and first evaluation took place ion 2003.	Medical officers, nurses and lab technicians	Maternal and Neonatal Health Care project personnel	Bangladesh Medical College Hospitals	Employment and retention	Natural deliveries increased by 63%, admissions of complicated cases increased by 135%, and cesarean deliveries increased by 70%.	Per trainee costs were approximately $1 550 for 1 year for MO, $1 020 for the 17-week competency-based team training, $340 for nurses, and $140 for laboratory technicians.	1Y, 2Y, 3Y, 4Y, 5Y, 6N, 7Y, 8Y, 9N, 10U, 11Y, 12Y
							Management			
							Monitoring and evaluation			
McCord [39] Tanzania prospective (before/after)	Trained AMOs to do cesarean sections and other emergency surgeries since 1963.		Tanzania started to train in 1963. Evaluation was done in 2006.	Assistant medical officers	Ministry of health			Among 1 134 complicated deliveries and 1 072 major obstetric operations, there was no significant difference between AMOs and MOs in outcomes, risk indicators, or quality.		1Y, 2U, 3U, 4Y, 5Y, 6Y, 7U, 8Y, 9N, 10Y, 11U, 12Y
Ohnishi [40] Paraguay prospective (before/after)	Comprehensive community-based ANC program	9 days. regarding maternal health care services, including comprehensive ANC programs, also involved hands-on practice	The pretest in 1997. Follow-up test in 1998. A post evaluation of follow-up test in June, 1999	Health care personnel (nurses, auxiliary midwives, and auxiliary nurses)	Physicians and nurses	Caazapa Regional Hospital		The average scores of the participants’ knowledge increased significantly from 41.0 before to 60.1 after training (*P* < 0.001). The enrollment rates of pregnant women in ANC increased from 2.2 times per pregnancy in 1996 to 3.4 times in 1998 (*P* < 0.001).		1Y, 2Y, 3Y, 4Y, 5Y, 6Y, 7Y, 8Y, 9N, 10Y, 11U, 12Y
Rana [41] Nepal prospective (before/after)	Comprehensive EmOC specifically for C-section and other surgical procedures was provided to junior doctors. BEmOC and post abortion care to nurses, as well as anesthetic services to nurses, health assistants, and senior auxiliary health workers	Varied from 5 days to 6 months depending on the type of training	Started in 2000 and the first assessment was done in 2001 and the program lasted for 4 years till 2004.	Doctors, nurses, AWH, ANM, medical officers, lab technicians, peons	Senior doctors used clinical training and curriculum for EmOC developed by JHPIEGO and AMDD	Hospitals	Infrastructure improvements	In 5 years, 3 comprehensive and 4 basic EmOC facilities were established in an area where adequate EmOC services were previously lacking. From 2000 to 2004, met need for EmOC improved from 1.9% to 16.9%; the proportion of births in EmOC project facilities increased from 3.8% to 8.3%; and the case fatality rate declined from 2.7% to 0.3%.	Technical training US$ 205 660	1Y, 2Y, 3Y, 4Y, 5Y, 6U, 7Y, 8Y, 9N, 10N, 11Y, 12Y
							Data collection			
									Management training US$ 97 170	
							Equipment			
							Policy advocacy and community information activities			
Population council [42] Ghana quasi-experimental	Self-paced learning (SPL) course and the 3-week residential course. Both courses covered theoretical and clinical training in life-saving skills, obstetric and infant care, family planning counseling, and post abortion care.	40 providers (midwives and physicians) in the experimental group received 6 months of SPL and a 1-week residential training course. In the comparison group, 35 providers attended the 3-week residential course.	Implantation started in 2001 and continued till 2004. Analysis was done during this period.	Midwives and physicians		2 administrative regions in northern Ghana		Knowledge improved in (SLP) group following the intervention, while clinical performance improved in both groups, with the residential group performing slightly better. Mean scores for management of obs complications, PAC, and pregnancy-related complications improved significantly in the SPL group.	The self-paced learning approach cost more per learner than the residential course (US$ 2 154 versus US$ 1 330).	Selection = UR
										Performance and detection = UR
										Attrition = UR
										Reporting = LR
Vaz [43] Mozambique quasi-experimental	Assistant medical officers with previous experience of surgical work were trained for 3 years.	3 years	The AMOs were trained in 1992, and the evaluation took place in 1996.	Assistant medical officers	Ministry of health			No difference in indication for cesarean deliveries. The only significant difference was in the group of superficial wound separation which was slightly more (0.35% vs 0.05%) in AMO vs specialist group.		Selection = UR
										Performance and detection = UR
										Attrition = UR
										Reporting = LR
Chilopora [44] Malawi prospective cohort study	COs were trained locally for 3 years.	3 years	The Government of Malawi has been training clinical officers since 1974.	Clinical officers	Government of Malawi		After a 1-year internship, they were licensed to practice independently.	No significant difference in postoperative maternal health outcomes, after emergency obstetric procedures performed by CO or by medical officers (RR 0.99; 95% CI, 0.95–1.03). No significant difference in stillbirth with procedures performed by CO (RR 0.75; 95% CI, 0.52–1.09) or in early neonatal death (RR: 1.40; 95% CI, 0.51–3.87). Although 22 maternal deaths occurred in 1 875 procedures performed by CO compared with 1 in 256 procedures performed by medical officers.		1Y, 2Y, 3Y, 4Y, 5U, 6U, 7Y, 8Y, 9Y, 10N, 11U, 12Y

To improve access to health care and conserve scarce health resources, some countries have trained mid-level providers and other cadre of health workers to deliver health care services. A study from Turkey and the Philippines compared intrauterine device (IUD) insertion by auxiliary nurse midwives (ANMs) with physicians at teaching hospitals and found no difference in those who were referred to a specialist after insertion of IUD (RR 0.93; 95% CI, 0.45–1.90) [31]. Similarly, in Thailand, a study compared postpartum tubal ligation by midlevel health workers and found no difference between the groups in postoperative complications (RR 2.43; 95% CI, 0.64–9.22) [30]. In a study from Nepal, the effectiveness of administration of early medical abortion by certified nurses and auxiliary nurses was compared with doctors [33]. Similarly, in South Africa and Vietnam, they compared manual vacuum aspiration (MVA) in women performed by an ANM or doctor’s assistants [32]. No significant difference in the likelihood of an incomplete abortion between groups of patients managed by ANMs and those managed by doctors (RR 0.93; 95% CI, 0.45–1.90) was found. No differences were found in the likelihood of a complication during (RR 3.07; 95% CI, 0.16–59.1)—or an adverse event after (RR 1.36; 95% CI, 0.54–3.40)—the MVA.

In Malawi [44], clinical officers (COs) were trained locally to perform major emergency and elective surgery. The study found no difference in postoperative maternal health outcomes, such as fever, wound infection, need for re-operation, and maternal death, when procedures performed by CO were compared to medical officers (MOs) (RR 0.99; 95% CI, 0.95–1.03). No difference was observed in stillbirth (RR 0.75; 95% CI, 0.52–1.09) or neonatal death (RR 1.40; 95% CI, 0.51–3.87). However, maternal deaths were 1.17% and 0.39% in the CO and MO group, respectively.

A study from Ethiopia [34] discussed the national program “Save the Mothers Project” that focused on providing three rounds of 3-month teaching to train service providers (general physicians (GPs), midwives, and others) on life-saving procedures in obstetrics and reported an increase in total number of instrumental deliveries from 6% in 1998 to 23% in 2001 due to considerable increase in the admission of complications. Similarly, in Bangladesh [38], MOs were trained for a year in obstetrics and anesthesia, nurses in midwifery for 6 months, and lab technicians in safe blood transfusions for a period of 2 weeks. As a result of the acquired skills, natural deliveries in the districts increased to 63%, admissions of complicated cases increased to 135%, and cesarean sections increased to 70%. The cost per trainee was approximately $1550 for MOs, $340 for nurses, and $140 for laboratory technicians.

The cost of training and deploying trained assistant medical officers (AMOs) was also compared among physicians in Mozambique [37]. They found that 30-year cost for major obstetric surgery was approximately $39 for AMOs and $144 for physicians. Doubling the salaries of AMOs resulted in smaller but still substantial difference in cost per surgery. Similar results were obtained from a study conducted in Tanzania [39], which started training AMOs for cesarean sections and other emergency surgeries in 1963. As a result, the met need increased and case fatality rate (CFR) decreased. There were no differences in outcomes, risk indicators, or quality of care in obstetric operations performed by AMOs and MOs.

A different approach was taken in studies conducted in Mozambique [43]. AMOs with previous surgical experience were trained for 3 years and were compared to obstetricians [43]. The only significant difference observed between the two groups was in superficial wound separation due to hematoma, which was slightly higher in AMOs (0.35%) versus the specialists (0.05%). Similarly, Nigeria [36] in 1993 trained midwives and residents with principles and practices of EmOC and henceforth found a reduction in treatment interval from 3.7 h to 1.6 h, and the proportion of women treated in less than 30 min increased from 39% to 87%. CFR fell from 14% to 11%. The annual number of women with complication declined as well.

Nepal, on the other hand, trained doctors, midwives, and nurses on different aspects of basic and comprehensive EmOC services [41] and found a reduction in CFR from 3.0% to 0.3%. Similarly, in Paraguay [40], nurses, auxiliary nurses, and auxiliary midwives were trained on theoretical and clinical aspects of life-saving skills in obstetric emergencies and reported an increase in knowledge. A study from Ghana [42] provided 6 months of self-paced and 1-week of residential training to midwives and physicians and compared them to a similar cadre given 3-week residential training on life-saving skills, obstetric and infant care, family planning counseling, and post abortion care. The residential group performed slightly better; however, overall levels of knowledge and performance remained low.

### Policy implementation

We identified four moderate-quality [12, 45–47] prospective before/after studies (Table 5).

**Table 5:** Studies related to HRH intervention: policy

Study	Policy implemented	When	Areas implemented on	Outcomes	Quality of the study
Efendi [12] Indonesia program evaluation (before/after)	Doctors and dentists were assigned as temporary staff on contract basis for a certain time period under “Contracted staff” or Pegawai Tidak Tetap (PTT) policy. Similarly, in the Village Midwife Program scheme, midwives were assigned to rural areas. In addition to the PTT scheme, the Special Assignment Program for Strategic Health Workers was implemented which included nurses, sanitarians, nutritionists, and other health cadres as well.	1991	Remote and very remote areas (division based on geographical position, access to transportation and the social economy)	Both these programs made a significant contribution to improving the availability of health workers in remote areas. As a result, in 2010, only 17% of the 9 000 very remote health centers were without a doctor, compared with 30% of 8 000 health centers in 2006*.*	1Y, 2U, 3Y, 4Y, 5Y, 6U, 7N, 8Y, 9U, 10N, 11N, 12Y
Akashi [45] Cambodia prospective (before/after)	User fees introduced at a public hospital, the National Maternal and Child Health Center (NMCHC) of Cambodia	1997	MOH started discussions to improve health care financing and introduce user contributions in 1995 and initiated a user-fee pilot program in selected national health facilities in 1997.	After the introduction of user fees, revenue was retained by the hospital to improve the quality of hospital services. Consequently, the patient satisfaction rate showed 92.7%, and the number of outpatients doubled. The average monthly number of delivery of babies increased from 319 to 585 in the third year after the user-fee introduction, and the bed occupancy rate also increased from 50.6% to 69.7%. As patient utilization increased, hospital revenue increased. The generatedrevenue was used to accelerate quality improvement, to provide staff with additional fee incentives to compensate their low government salaries, and to expand hospital services.	1Y, 2U, 3Y, 4Y, 5Y, 6U, 7Y, 8Y, 9N, 10N, 11N, 12Y
Koblinsky [46] Bangladesh prospective (before/after)	In 1994, the EmOC approach dominated with assistance from the UNICEF, UNFPA, and the AMDD program in the renovation and up gradation of existing facilities and training of facility staff. With the development of the National Maternal Health Strategy in 2001, the approach broadened, building on the rights’ approach for safer motherhood and was incorporated into the ongoing Health and Population Sector Programme (HPSP) and subsequently into the Health, Nutrition and Population Sector Programme (HNPSP)	1994 and 2001 and first evaluation took place in 1995	EmOC at the facility level	Since 1990, the MMR in Bangladesh has declined from 514 in 1986–1990 to 400 in 2003—22% in the 11 intervening years.	1Y, 2U, 3Y, 4Y, 5Y, 6U, 7Y, 8U, 9N, 10N, 11U, 12Y
			CSBAs providing safe delivery care at home.		
				Deaths from induced abortion have declined when the 1995–2005 level is compared with the pre-intervention levels of 1976–1980.	
				During 2000–2004, MMR was 322; only 13% of delivering-women used professional care for birthing, and 9% of births were in facilities. By 2007, 18% were delivering with professional care and 15% were in facilities.	
				For cesarean section in rural areas, the rate increased from 0.9% to 1.7% from 1995–1996 to 2000–2004 and then to 5.4% in 2005–2007, while in urban areas, the corresponding rates doubled—from 5.6% to 11.4% and then increased to 16.2% in 2005–2007.	
				The increase in the use of antenatal care has shown promise—from 27% in 1991–1994 to 60% in 2005–2007.	
Rath [47] Nepal prospective (before/after)	The Nepal National Safer Motherhood Project was a collaborative intervention between the Nepal Ministry of Health and Population and the UK Department for International Development (DFID), managed by Options Consultancy Services.	1997–2004, evaluation was done yearly	In phase 1, the Project focused mainly on improving midwifery and emergency obstetric services in selected health facilities in 3 districts and then in phase 2, to 6 districts. Two main components were developed: (i) management of service provision for women of reproductive age, including improvements to the physical infrastructure of hospitals, equipment and supplies, and training of personnel and (ii) increasing access to midwifery and obstetric services by improving the social context to enable women to utilize services.	Availability of birthing facilities	1Y, 2U, 3Y, 4Y, 5Y, 6U, 7Y, 8Y, 9N, 10N, 11U, 12Y
				Met need for emergency obstetric care was <5% in the phase 1 districts in 1997. The average annual increase in met need has been 1.3% per year over the intervention period, bringing it to the 2004 level of 14% in public sector facilities in project-supported districts. In a further 4 districts supported by UNICEF, met need increased from 1.9% to 16.9% between 2000 to 2004.	
				Availability of a skilled birth attendant near the home	
				The 2001 Demographic and Health Survey (DHS) found that only 3.1% of deliveries of the approximately 900 000 births per annum were attended by an auxiliary nurse midwife or nurse. This had increased to 8.3% in the 2006 DHS.	
				Free or reduced costs for services and transport	
				Communities valued these funds and that they increased confidence in being able to cope with emergencies.	

In the beginning of 1994, Bangladesh upgraded its EmOC facilities and trained service providers in those facilities [46]. However, from the beginning of 2001, to complement the facility approach to obstetric care, a skilled birth attendant strategy was initiated with guidance from the World Health Organization and UNFPA. As a result, measles, mumps, and rubella (maternal mortality ratio (MMR)) declined to 22% in the 11 intervening years. Professional care increased from 13% to 18% and rates of cesarean section from 0.9% to 5.4%. The policy implementation also affected the antenatal consultation which doubled from 27% in 1991–1994 to 60% in 2005–2007.

Similarly, the Nepal National Safe Motherhood Project [47], implemented from 1997 to 2004, focused on improving emergency obstetric services and midwifery care in selected health facilities. Government policy was developed to increase access to midwifery and obstetric services to improve management of service provision for women of reproductive age by working with NGOs for training and service provision and non-state health workers. As a result, the average annual met need increased from 1.3% to 14%. Deliveries attended by ANMs or nurse increased from 3% in 2001 to 8% in 2006. Free or reduced cost for services and transport was valued by the communities, and the intervention increased their confidence to cope with emergencies.

In Cambodia, to compensate low wages, health care workers demanded unofficial payments from patients in facilities where health care services were provided free of charge. Introduction of a user fee at the national maternal and child health center of Cambodia [45] assisted the hospital to retain revenue and improve the quality of services focusing on the work environment and conditions of HR intervention. Patient satisfaction increased to 93%, and average monthly number of deliveries increased from 319 to 585.

The Indonesian government in order to overcome the shortage of health workers [12] implemented compulsory services in rural areas to support the recruitment and deployment of medical staff in remote and very remote areas. Doctors, dentists, and midwives were assigned as temporary staff on contract basis for a certain time. In addition, a special assignment program was implemented which included other health cadres as well where they received travel expenses and additional incentives for a period of service and according to the remoteness. Both these programs improved the availability of health workers in remote areas. As a result, in 2010, only 17% of the 9000 very remote health centers were without a doctor, compared with 30% of 8000 health centers in 2006*.*

### Combined interventions

We found six low- to moderate-quality prospective before/after studies [24, 48–52] with combined interventions which focused on HR training, policy and advocacy, partnerships, and supervision [49] (Table 6).

**Table 6:** Studies related to HRH intervention: combined interventions

Study	HRH management system								Others
	Training	Policy	Management	Incentive	Supervision	Partnership	Personnel system	Intervention to evaluation duration	
Kayongo [50] Peru (before/after)									
Implementation	Training sessions for 15 days with on-call duty after an analysis of the causes of maternal death, the treatment, and prevention of postpartum hemorrhage received special emphasis in the trainings.		Development of a more efficient mechanism for recordkeeping and data collection.		Quality of care was enhanced through the use of criterion-based audits. External supportive supervision and on-site quality improvement processes were used to enhance efficient service delivery.	The FEMME Project worked with community groups to form local committees. CARE’s most important partners in the FEMME Project have been the IMP in Lima, the Ayacucho DIRESA, and the Regional Hospital.		The intervention started in 2000 and the first evaluation took place in 2001 and then in next three years till 2004.	Facility setup, including adequate infrastructure, equipment, and supplies
			Placement of trained staff to ensure a wide distribution of technical capability to resolve obstetric emergencies.						
Outcomes	CFR decreased from 1.7% to .01%, increase in met needs from 30% to 84% in 5 years, and a small increase in cesarean sections from 4% to 6%.								
	Quality: 1Y, 2Y, 3Y, 4Y, 5Y, 6Y, 7Y, 8Y, 9N, 10N, 11U, 12Y								
Kayongo [51] Rwanda (before/after)									
Implementations	CARE conducted several trainings to provide doctors and midwives to manage major obstetric complication. Most significant training course was a 12-module competency-based training.		Staff, including doctors and midwives, were trained and supported to ensure complete recording of case notes and filling out of registers.		Main strategies of the project were to engage the participation of district supervisors as partners for improving and transforming this process.	Stakeholders in the MoH, local partners in safe motherhood such as UNFPA, district health officials, and hospital health professionals were involved in various process of the project.		The interventions started in 2001 with first evaluation in 2002 and then consequently in 2003 and 2004.	Renovations and provision of essential equipment and supplies
Outcomes	Numbers of deliveries increased by almost 25% from 2001 to 2002, and the obstetric complications managed increased by almost the same magnitude (26.5%). Cesarean section increased by 63% during this time. There was a continuous decrease in the case fatality rate over the 4 years of the project from 2.2% in 2001 to 1.8 in 2002 and finally 1.2% in 2004.								
	Quality: 1Y, 2Y, 3Y, 4Y, 5Y, 6Y, 7Y, 8Y, 9N, 10N, 11U, 12Y								
Jamisse [49] Mozambique (before/after)									
Implementations	Technicians trained in surgery and anesthesia, nurses trained as surgical assistants. MNCH nurses and midwives were trained in basic and comprehensive EmOC and management of major obstetric complications.				Supervision of the activities was the responsibility of the Ministry of Health.			Intervention started in 1998 and the first evaluation was done in 1999 and then consequent evaluations for 2 more years.	Supplementing equipment and essential supplies at the EmOC units
									Radio communication and transport system was established
Outcomes	José Macamo Hospital, which dealt with 14% of all deliveries and 2.5% of all C-sections in 1998, was responsible for 32% of all deliveries and 38% of all C-sections in Maputo city in 2001. Mavalane never succeeded in providing comprehensive EmOC 24 h a day. It did succeed, however, in almost doubling the number of deliveries, from 2 500 in 1998 to almost 5 000 in 2001. While in 1998 the Manhica Hospital managed 29% of institutional deliveries and 8.2% of cesarean sections in the district, these percentages increased to 33% and 31.2%, respectively, in 2001. The maternal deaths per total number of deliveries occurring in the district’s institutions were 572/100 000 live births in 1998 and 433/100 000 in 2001. The case fatality rate in basic EmOC units decreased from 4.7 in 2000 to 2.4 in the first 6 months of 2002								
	Quality: 1Y, 2U, 3U, 4Y, 5Y, 6U, 7U, 8Y, 9N, 10N, 11U, 12Y								
Santos [52] Mozambique (before/after)									
Implementations	The 4-week training session for basic EmOC consisted of 1 week of theory and 3 weeks of practical hands-on experience, emergency transport, and referral system.	Policy clearly endorsed EmOC	The project used the UN process indicators for obstetric services as its monitoring tools		The Medical Director of the Provincial Health Directorate and the Chief Nurse were given the responsibility to coordinate all activities of the project, which included frequent supervisory visits to the facilities.	AMDD’s partner in Mozambique was UNFPA. AMDD was supported by the Bill and Melinda Gates Foundation.		Interventions started in 1999, and first evaluation began in 2002 and was continued for 3 years till 2005.	Renovation of the hospitals, equipments and emergency drugs and supplies were provided
Outcomes	Utilization among women with complications (met need or the proportion of women expected to have complications who are admitted for treatment) increased 3-fold, from 11.3% to 32.8% in all facilities. The aggregate case fatality rate (CFR) was reduced by almost half (2.9% to 1.6%).								
Islam [48] Bangladesh (before/after)									
Implementations	Training of medical officers was originally designed as a 6-month course but was later extended to 1 year. Training of nurses was extended from 6 weeks to 4 months. Laboratory technicians participated in a 2-week training course.		A checklist was developed for monitoring visits to training facilities to capture information such as trainees’ performance, lecture classes, opportunities for skills practice, training facility caseload, number of other trainees in the department, training problems, and general observations recorded in reports.	Trainees were provided with a monthly scholarship, book grant, travel allowance, and training materials.	Training activities were coordinated locally by the Training Coordination Committee at each medical college hospital.	UNFPA and UNICEF	Manager of the Directorate General of Health Services selected the medical officers for training, while nurses and laboratory technicians were selected from the facilities.	Intervention started in 2003 and evaluation was done in 2004.	Supply of necessary equipment and logistics. Renovations of the facilities
Outcomes	In 2004, 105 of the 120 sub-district hospitals had become functional for EmOC, 70 with comprehensive EmOC, and 35 with basic EmOC, while 53 of 59 of the district hospitals were providing comprehensive EmOC compared to 35 in 1999.								
	Quality: 1Y, 2Y, 3Y, 4Y, 5Y, 6Y, 7Y, 8Y, 9N, 10N, 11U, 12Y								
Barker [24] Nepal (before/after)									
Implementations	Ongoing work to incorporate training for skilled birth attendants into pre-service courses for doctors and nurses	SSMP worked with other safe motherhood stakeholders		SSMP supported Maternity Incentives		Civil society, political parties, local media, development program, and health workers. Provided technical and strategic planning support for training		Interventions started in 1997, and evaluations began in 1998 and continued till 2005 every year.	Supplies of emergency drugs and equipment
Outcomes	Utilization of antenatal care services increased from 39% to 72%, delivery by a trained health worker from 9% to 19%, institutional delivery from 8% to 18%, and cesarean sections from 1% to 2.7%. CFR decreased from 0.5% to 0.4%.								

In one study from Rwanda [50], CARE’s work supported a comprehensive package of interventions which included training of the doctors and midwives to manage major obstetric complications. As a result, numbers of deliveries and management of obstetric complications increased by almost 25% and 27%, respectively, from 2001 to 2002. A continuous decrease in the CFR was also observed from 2.2% in 2001 to 1.8% in 2002 and finally 1.2% in 2004.

Similarly, in Peru [51], a decline in CFR from 1.7% to 0.1% and increase in met needs from 30% to 84% were observed after the implementation of a 15-day training and supervision program to improve the quality of care. Likewise, Mozambique [52] trained 137 different professionals and adopted an enhanced policy for the improvement of the quality, access, and utilization of the EmOC such as supportive supervision, logistics for supplies, provision of equipment and drugs, record keeping, monitoring and evaluation, and quality improvement techniques. Maternal death audits showed an increase in met needs from 11% to 33% and decrease in CFR from 3% to 1.6%. Similarly, in Nepal [24], utilization of antenatal care services increased from 39% to 72%, delivery by trained SBAs from 9% to 19%, institutional delivery from 8% to 18%, cesarean sections from 1% to 3%, and CFR decreased from 0.5% to 0.4% after the establishment of policy to provide EmOC to the most deprived population. Incentives were given to the mothers and health workers to address the financial barriers to women accessing maternal services. A range of external agencies supported staff training; infrastructure and equipment; behavior change interventions promoting antenatal, skilled delivery, and postpartum care; and community emergency funds and transport schemes.

## Discussion

A major part of this review was aimed at exploring how HRH interventions lead to improved maternal health outcomes, and we also came across studies with successful HRH strategies pertaining to general health outcomes [12, 16, 18, 53]. The findings, although mostly from low- to moderate-quality studies, showed that HRH interventions can contribute positively to the health worker’s performance and improved maternal outcomes. However, we still feel that HRH interventions in relation to maternal health are not widely researched.

**Fig. 3:**
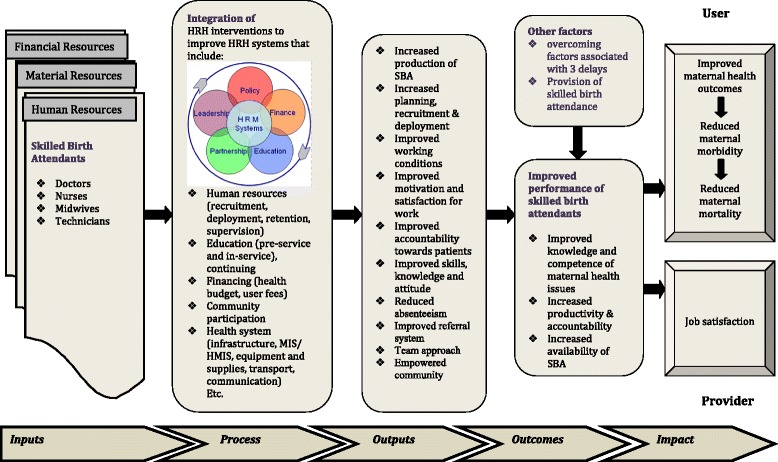
Conceptual framework of HRH interventions for improved maternal outcomes

The WHO framework (Fig. 1) shows the steps required for improving general health outcomes; we developed a framework to facilitate understanding of mechanisms, based upon dimensions of health worker performance (Fig. 3), to explain the effect on maternal mortality. It reveals a variety of interrelated mechanisms which can lead to improved health worker performance and maternal health outcomes provided other associated or confounding factors are addressed. Implementation of a HRH management system to improve the availability, training, education, and retention of doctors, nurses, midwives, and technicians is one of the factors contributing to improved maternal health. Other components included in the studies were supervision and partnerships which improved the health system effectiveness and hence maternal health. Increase in knowledge and competence with concomitant increase in accountability and productivity of SBAs will lead to better health outcomes resulting in decrease in maternal mortality and morbidity.

The review of 25 studies revealed certain reasons why maternal mortality and morbidity is still high in LMICs despite the efforts and policies implemented throughout these years. It is observed that in many LMICs some components of the HRH intervention were applied with positive effects on the maternal health and health care delivery to the rural areas. When a professional (doctor or midwife) is linked up with a strong referral system, maternal health outcomes improve and mortality decreases significantly irrespective of whether the birth took place at home or in a health facility [54]. Programs should therefore integrate maternal health and family planning as decline in fertility can reduce maternal mortality which can be achieved by aligning formal/informal linkages or partnering different programs [55]. Secondly, it is important to educate women about their health and complications during pregnancy, and evidence suggests that awareness improves care seeking [55, 56]. However, poverty is one of the main factors affecting the access to maternal health care in rural areas which warrants combating gender inequalities [56, 57]. In such scenarios, community involvement has been found to be integral in raising the awareness in community which can further be scaled up to integrate micro-financing schemes to help improve the financial stability in families/community.

In the review, we found that only few components of the HRH management system were investigated. Most of the studies explored the impact of training and continuing education of the health care providers on maternal health individually or as combined intervention. However, less or no work was found on the HRH systems studying the impact of recruitment, employment, hiring, deployment, migration, health safety, and retirement on maternal health outcomes. Similarly, no study was found on the financial component addressing the budget allocation for salaries and allowances, education and training, and HRH expenditure data. Gaps were identified in other HRH interventions related to staff motivations such as job satisfaction, appraisals, workplace safety, career development, and improvement in the work environment as well as the HRH information system. Moreover, studies that reported the impact of training SBAs on improved health outcomes rarely reported if those results were merely because of training alone or there was a role of other related co-interventions as well.

Improvements in HRH are required to be made by LMICs to raise the awareness among women for health care seeking from SBAs and at the same time improving the delivery of health care. The need for a multi-sectoral approach to women health is emphasized. This includes the training of staff, their employment and retention, and improved management systems in the formal health care [58]. It is important to address the keen desire of continued education/training of health workers especially doctors. The training and education programs implemented in some countries need to be taken on the larger scale. Also, the keen desire of health workers especially doctors embarking higher positions should be addressed. Government should work in conjunction with other stakeholders and donors to form policies for education and finance and develop health care models to suit the needs of the countries in managing EmOC and decreasing maternal mortality.

We recommend that more studies need to be conducted on the other HRH management system components such as supervision, partnership, and proper HRH information system development especially in the context of SBAs and their impact on the maternal health. Financial constraint remains the most important barrier to achieve the targets. Therefore, interventions targeting the budget allocations for the salaries and education of the health care providers need to be implemented and their impact on maternal mortality to be studied. It is well documented that more lives of mothers can be saved if adequate importance is given to the EmOC services and if they are made an integral part of the health system [59, 60]. The importance of collaboration between SBAs and other health care providers like obstetricians and anesthesiologists as well as lay health providers has also been emphasized [25, 61], and its integration in national policies has also been underscored for better outcomes [58, 62–64]. Political sensitization is needed at the local level, particularly with local policies [65]. Investment at all levels to strengthen the comprehensive EmOC and family-planning program is required [55]. Political parties, stakeholders, and donors need to work together to invest in improving the availability and quality of maternal care services [56]. A high level of political commitment is also required in investing, developing, deploying, and supporting a cadre of health providers with midwifery skills [66].

It is important to assess and urgently address the barriers to recruitment, deployment, and retention of skilled personnel in LMICs [67] and to discourage the process of “brain drain” [68, 69]. The deployment, recruitment, and retention of care providers especially nurses and specialists are a major challenge on the supply side [69–71].

Furthermore, it is important to provide health care professionals with ongoing training [72, 73]. A study from Kenya reported that only 18% of the staff had received life-saving skills and only 37% received training related to prevention of mother-to-child HIV transmission, which is the utmost required skill in a country like Africa [73]. Thus, it emphasizes the importance of training to meet the needs of the population.

The limited management capacity is one of the main reasons of slow progress in maternal health [71]. It is observed that a well-functioning health system with appropriate supply of equipment, drugs, and other supplies is required for timely management of delivery complications to prevent maternal deaths [2]. Studies found that the utilization of health services may be low because of the gender inequality and status of the women as well as cultural barriers. Empowerment of women and education has shown to positively influence the health-seeking behavior and decrease in maternal mortality [74–77]; thus, efforts should be made to improve this area for sustainability of the interventions to decrease maternal mortality and improve overall health care utilization.

It is seen that HRH interventions alone cannot improve the functioning and availability of the skilled health workers. There are many other areas to gain the best outcome regarding maternal health. Female education, empowerment, and gender equality should be emphasized [62]. Effective provision of services can only be made possible when huge inequalities in maternal care are removed from the community. The poor-rich inequalities in delivery care can be reduced by providing equal services to all by rigorous efforts made towards equity oriented research, policy making, implementation, and proper monitoring of the services provided [57]. Investment in social and economic development with emphasis on achieving gender equality should be the goal [66]. Similarly, access and availability of affordable transportation in rural areas as well as appropriate supply of drugs, equipment, and infrastructure of the health facilities are needed to improve EmOC and reduce maternal mortality.

## Conclusions

This review suggested an urgent and immediate need for formative evidence-based research on effective HR interventions for improved maternal health in LMICs. The principal challenges in these countries are to strengthen research systems, identify key research questions, and generate the capacity to turn research into practical applications [78]. The studies showed that all the HRH interventions implemented individually or in combination had a positive impact on improving maternal health. However, implementation alone is not enough to bring about this change but also other essential steps like educating and empowering women, alleviating poverty, establishing gender equality, and providing infrastructure, availability of equipment, drugs, and supplies are all an integral part of working towards the achievement of SDGs and reducing maternal mortality. Leaders and stakeholders are required to work together as a team to construct new models of health care according to the individual needs of each country.

## Abbreviations

AMO: assistant medical officer
ANM: auxiliary nurse midwife
CFR: case fatality rate
CO: clinical officer
EmOC: emergency obstetric care
GP: general physician
HRH: human resources for health
LMICs: low- and middle-income countries
MDG: Millennium Development Goal
MO: medical officer
MVA: manual vacuum aspiration
RCT: randomized controlled trial
SBA: skilled birth attendant
